# Risk of malignancies among asymptomatic postmenopausal women with thickened endometrium: A cohort study

**DOI:** 10.1097/MD.0000000000014464

**Published:** 2019-02-08

**Authors:** Zhe Li, Lei Li

**Affiliations:** a Department of Obstetrics and Gynecology, Peking Union Medical College Hospital; b Department of Gynecologic Oncology, National Cancer Center/National Clinical Research Center for Cancer/Cancer Hospital, Chinese Academy of Medical Sciences and Peking Union Medical College, Beijing, China.

**Keywords:** endometrial cancer, hysteroscopy, menopause, transvaginal sonography

## Abstract

The risk of malignancies and related factors among asymptomatic postmenopausal women with thickened endometrium in transvaginal sonography (TVS) are unclear.

In this longitudinal study at a tertiary teaching hospital, all medical records of hysteroscopy were searched and retrospectively reviewed according to age, TVS results and diseases coded as International Classification of Diseases version 10. Asymptomatic postmenopausal women with endometrial thickness ≥5 mm from January 2006 to January 2016 were included. A follow-up was provided up to January 2017.

Four hundred eighty-eight patients were included with a median endometrial thickness of 8 mm (range 5–30) in TVS. The most common pathologic findings were polyps (51.0%) and normal endometrium (34.2%). Fifteen (3.1%) and 10 cases (2.0%) had endometrial intraepithelial neoplasia (EIN) and carcinoma. Patients with carcinoma had significantly more abnormal serum CA125, thicker endometrium, and more lesions with positive Doppler flow signals. In receiver operating characteristic curve analysis, endometrial thickness of 12 mm had the best predictive ability for malignancies. In multivariate analysis, endometrial lesion with positive Doppler flow signals in TVS was the only independent factor for EIN/carcinoma (odds ratio [OR] 8.0, 95% confidence interval [CI] 1.4–45.1) and for carcinoma (OR 16.0, 95% CI 1.3–192.8). After a median follow-up of 45 months, carcinoma occurred in 1 of 35 (2.8%) women with repeated thickened endometrium.

Among asymptomatic postmenopausal women with thickened endometrium, the risk of EIN and malignancy was minimal but worth of long-term follow-up. Endometrial lesions with positive Doppler flow signals in TVS suggested a high risk of malignancy.

## 1 Introduction

Uterine carcinoma ranks tenth and twelfth in new cancer cases and deaths among Chinese female cancers.^[1]^ During 2004 to 2010, 5-year overall survival of uterine corpus reached 83% in America^[2]^ About 90% endometrial cancer patients had experienced vaginal bleeding, while an asymptomatic malignancy may occur in less than 20% of patients.^[3,4]^ For patients with postmenopausal vaginal bleeding, endometrial thicknesses measured by transvaginal sonography (TVS) were significantly correlated with risk of endometrial cancer.^[5]^ Among asymptomatic postmenopausal women with a thickened endometrium (traditionally defined as ≥5 mm), studies stated a malignancy rate 0% to 3%.^[6,7]^ For the reasonable cut-off value of endometrial thickness, scholars once recommended various criterion based on their experiences rather than evidences.^[8]^ Primary aim of this study is to explore the risk of endometrial malignancies and related factors among asymptomatic postmenopausal women with endometrial thickness ≥5 mm. We also tried to find out a cut-off value of endometrial thickness to predict malignancies in such situation.

## 2 Material and methods

### 2.1 Study design and sample size

This study was conducted at Department of Obstetrics and Gynecology, Peking Union Medical College Hospital (PUMCH), performed as part of the study of “Survival Outcomes of Uterine Malignancies in Chinese Population”. Institution Review Board of PUMCH has approved this study (ZS-1428), and the registration number at *clinicaltrials.gov* is NCT03291275 (SOUM-1). All patients presented their consents before hysteroscopy.

With class I and class II error probability (*α* and *β*) of 0.05 and 0.20, based on 1%^[9]^ and maximum of 3%^[10]^ incidence of malignancies in general postmenopausal women and postmenopausal women with endometrial thickness ≥5 mm, at least 572 cases with definite pathologic outcomes are needed to find out significance of cancer incidences.

### 2.2 Participants and follow-up

All medical records of hysteroscopy between January 2006 and January 2016 in PUMCH were searched and retrospectively reviewed according to age, TVS results and diseases coded as International Classification of Diseases version 10.

Eligible patients were included if:

(1) natural menopause was confirmed of no less than 1 year,
(2) hysteroscopy was performed for the thickened endometrium (≥5 mm) in TVS;
(3) last TVS was performed within 1 week before hysteroscopy, and there was no obvious adnexal mass;
(4) patients had no symptoms of postmenopausal vaginal bleeding, abnormal vaginal discharge or fluids, or lower abdominal pain.

Exclusion criteria consisted of patients with an unknown status of menopause or with any aforementioned symptoms, or patients treated with only dilation and curettage (D & C) without hysteroscopy.

All patients were followed up to January 2017. The diagnosis of recurrence of endometrial diseases was confirmed by reviewing medical records.

### 2.3 Transvaginal sonography

The endometrial thickness was measured by TVS as the thickest part in the sagittal plane of the uterus and recorded as a single-layer endometrial thickness excluding cavity fluid. Last TVS must be performed within 1 week before hysteroscopy to assure the diagnosis of thickened endometrium. The adnexa was also examined by TVS. Endometrial lesions which were defined as different echogenicity occupying in the uterine cavity and related Doppler flow signals were reviewed especially.

### 2.4 Data collection

Epidemiological and clinicopathologic data were retrospectively collected from medical records by Dr Z Li, and checked by Dr L Li. We gave special concerns to the data of age of hysteroscopy, postmenopausal periods, body mass index (BMI), serum CA125, history of cancer and medicine usage (menopausal hormone treatment [MHT] and tamoxifen). Serum CA125 was classified as normal (<35 U/ml) and abnormal (≥35 U/ml) values. All specimens from hysteroscopy were reviewed by pathologists, and the discoveries were classified as benign, atypical hyperplasia (or endometrial intraepithelial neoplasia [EIN]) and endometrial carcinoma. Complications of hysteroscopy were recorded according to Common Terminology Criteria for Adverse Events v4.03.^[11]^

### 2.5 Statistics

Statistical analyses were performed with SPSS version 20.0 (SPSS Inc, Chicago, IL). Potential confounders were identified using the nonparametric *κ*^2^ test or Fisher exact test and Mann–Whitney *U* test. Multiple parameter analyses were performed using binary logistic analysis calculating odds ratios (OR) and 95% confidence intervals (95% CI) with all the parameters having significances in univariate analysis. Receiver operating characteristic (ROC) curve analysis was used to find the cut-off value of endometrial thickness for EIN and carcinoma by area under curve (AUC).

## 3 Results

### 3.1 Characteristics of patients and surgeries

**Figure 1:**
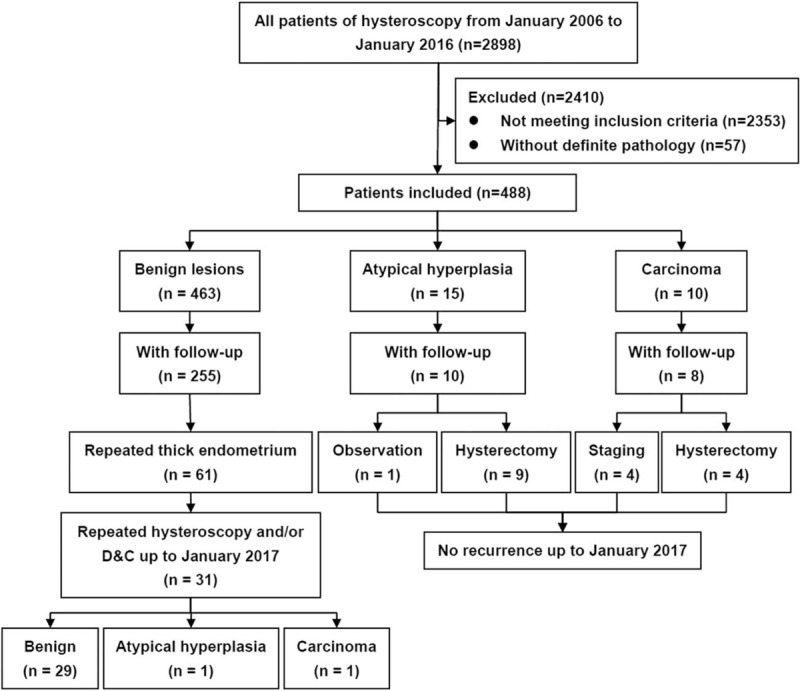
Flow diagram of the study.

From January 2006 to January 2016, among 2898 patients of hysteroscopy, 488 eligible patients were included (Fig. 1). All patients had definite endometrial thickness ≥5 mm in TVS within 1 week before hysteroscopy. Average age and BMI were 60.1 ± 7.0 years and 25.0 ± 3.8 kg/m^2^ respectively. Median duration of menopause period, gestation and parity were 8 years (range 1–38), 2 (range 0–10), and 1 (range 0–4). Before hysterectomy, 14 of 292 (4.8%) patients had abnormal serum CA125, and 68 (13.9%) patients had accepted various regimens of progesterone. There were 59 (12.1%) and 22 (4.5%) patients with a history of breast cancer and colorectal cancer, respectively, 21 (4.3%) and 31 patients (6.3%) with a history of MHT and tamoxifen treatment, respectively. In TVS, the median endometrial thickness was 8 mm (range 5–30), 29 (5.9%), and 96 (19.7%) patients had fluid and endometrial lesions in uterine cavity respectively. For 96 patients with endometrial lesions, 13 (13.5%) had positive Doppler flow signals.

Among 488 eligible patients, 10 cases (2.0%) of perforation of uterus and 1 case (0.2%) of perforation of bowel occurred during hysteroscopy. No other severe adverse events happened. All patients recovered uneventfully in the end.

### 3.2 Pathologic outcomes

Pathologic outcomes of biopsy from endometrium were listed in Table 1. There were 463 cases (94.9%) of benign results: 249 cases of endometrial polyps, 167 of normal endometrial tissue, 12 of leiomyoma, 13 of hyperplasia, and 22 of blood clot or mucus. Fifteen (3.1%), and 10 cases (2.0%) had EIN and carcinoma.

**Table 1:**
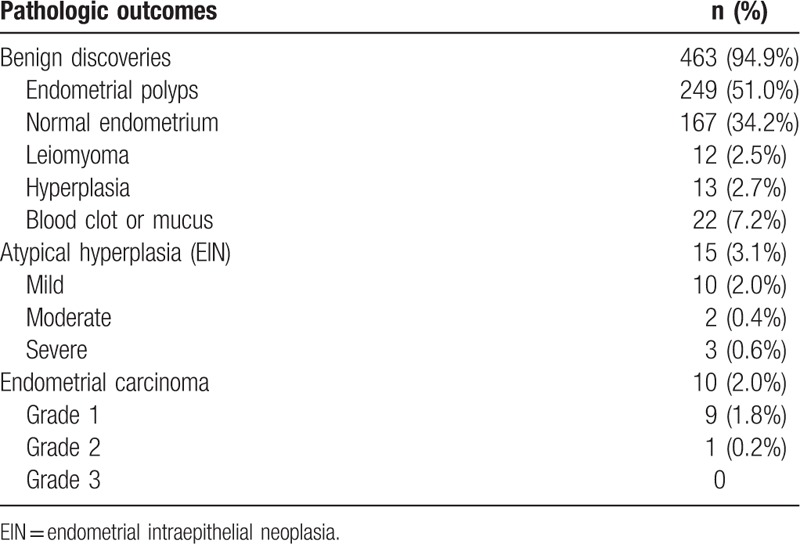
Pathologic outcome in 488 menopausal asymptomatic women with endometrial thickness ≥5 mm.

### 3.3 Cut-off value of endometrial thickness for predicting malignancies

In ROC analysis, endometrial thickness of 12 mm had maximum AUC (0.716, 95% CI 0.534–0.897, *P* = .019) for differentiating patients with benign lesions and malignancies, and endometrial thickness of 11 mm have maximum AUC (0.587, 95% CI 0.465–0.708, *P* = .144) for differentiating with benign lesions and EIN/malignancies.

### 3.4 Risk factors of EIN and/or endometrial carcinoma

Compares between patients with benign results and EIN/carcinoma, and between patients with benign results and carcinoma are listed in Table 2. There were no significant differences about age, gravidity, parity, BMI, postmenopausal duration, serum CA125, medical history of cancer, hormone usage, fluid in the uterine cavity or endometrial lesion in TVS. In univariate analysis, patients with EIN/carcinoma had more abnormal CA125 values (*P* = .047) and more endometrial lesions with positive Doppler flow signals (*P* = .031); while patients with carcinoma had more abnormal CA125 values (*P* = .047), more thicker endometrium (*P* = .007), higher proportion of endometrial thickness ≥12 mm (*P* = .013), and more endometrial lesions with positive Doppler flow signals (*P* = .043). In Logistic regression model, endometrial lesions with positive Doppler flow signals was the only independent factor for EIN/carcinoma (OR 8.0, 95% CI 1.4–45.1, *P* = .018), and for carcinoma (OR 16.0, 95% CI 1.3–192.8, *P* = .029), while abnormal CA125 or endometrial thickness had no predictive values for the risk of EIN and/or endometrial carcinoma.

**Table 2:**
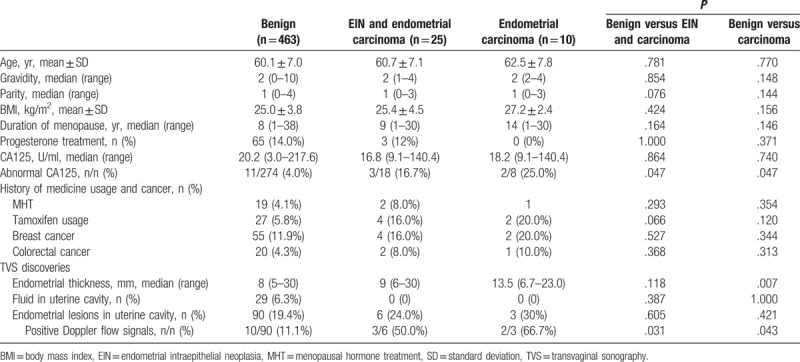
Demographic and clinical characteristics of patients with different pathologic outcomes.

### 3.5 Follow-up

Among 463 patients with benign discoveries, 255 cases (55.1%) were followed up to January 2017 with a median follow-up period of 45 months (15–108). During follow-up, 61 patients had repeated thickened endometrium in TVS, of which 35 cases accepted another hysteroscopy and/or D & C, which ultimately discovered 1 case of EIN and 1 case of endometrial carcinoma.

Among 15 patients with EIN, 10 cases (66.7%) were followed up with a median follow-up period of 44 months (23–105). One patient accepted long-term observation without further surgery and was free of progression. Nine patients accepted single hysterectomy, and 5 had no EIN left, 2 had EIN in situ, and 2 had G1 endometrioid carcinoma within endometrium without invasion of the myometrium in final specimens.

Among 10 patients with carcinomas, 8 cases (80.0%) were followed up with a median follow-up period of 56 months (35–108). Four and 4 patients accepted single hysterectomy and complete staging. All patients belonged to stage IA according to the classification system of the International Federation of Gynecology and Obstetrics. There was no recurrence till the end of follow-up.

## 4 Discussion

In 2001, American Cancer Society concluded that there was insufficient evidence to recommend screening for endometrial cancer in women at average risk or increased risk because of a history of unopposed estrogen therapy, tamoxifen therapy, late menopause, nulliparity, infertility or failure to ovulate, obesity, diabetes, or hypertension.^[12]^ At the time of menopause, women should be informed about the risks and symptoms of endometrial cancer and strongly encouraged to report any unexpected bleeding or spotting to their physicians.^[13]^ Even for women with a mismatch repair gene mutation, there was no statistically significant association between endometrial cancer and age at first and last live birth, age at menopause, and postmenopausal hormone use.^[14]^ Similarly, hormone usage and cancer history had no impact on endometrial cancer risk in our study. In univariate analysis, abnormal CA125 suggested a higher risk of cancer, but the significance was limited due to small sample size and insignificance in multivariate analysis.

Risks of malignancies or EIN were minimal among asymptomatic postmenopausal women in previous reports^[6,15–17]^ and in our study. It is questioned whether endometrial thickness was a sole indication of surgical intervention in asymptomatic postmenopausal women.^[18]^ Pool analysis,^[17]^ meta-analysis,^[18]^ and prospective study^[19]^ all failed to find out proper cut-off value on endometrial thickness in asymptomatic postmenopausal women, as well as in our study. On the other hand, universal hysteroscopy for asymptomatic women would cause unnecessary interventions and severe complications 0.95% to 13.6% according to previous reports.^[7,20,21]^ Uterine perforation with bowel damage occurred in 2 of 54 (3.7%) women who underwent saline contrast sonohysterography survey,^[22]^ and occurred in 2.0% patients of hysteroscopy in our patients. Even for patients with malignancies in our cohort, the clinicopathologic characteristics and prognosis were relatively favorable, and previous studies did not ascertain a significant difference in prognosis between asymptomatic and symptomatic patients.^[23–25]^ These findings would provide efficient discussion and decision-making with patients, thereby probably reduce plenty of invasive interventions and anxiety derived from fear of risk of cancer.

Although there is no established screening method for endometrial cancers among the general population, TVS could provide details of endometrial changes with a high agreement with pathology, especially among symptomatic women.^[26,27]^ Our study shows that endometrial lesions with positive Doppler flow signals prompt non-benign pathology and endometrial carcinoma. However, Goldstein reported that there was no association between Doppler flow, resistive index (RI), or pulsatility index (PI) and the risk of cancer in a study of 61 women with polyps.^[28]^ Lieng found that there were no significant differences in PI and RI before enhancement by contrast between women with endometrial polyps and those with endometrial cancer.^[29]^ All these studies did not take into the issues of endometrial thickness or menopausal status, and the sample sizes had no enough weight to draw a conclusion, hence the role of TVS parameters deserve further prospective analysis. In addition, it has been reported 1.5% (25/1654) patients had atypical hyperplasia or endometrial carcinoma among asymptomatic postmenopausal women,^[30]^ and endometrial polyps were associated with an increased risk of endometrial malignancy.^[30–33]^ It was still unknown whether de novo polyp development was estrogenic-driven in postmenopausal women, which can also induce carcinomatosis.^[34]^ If the stimulation leading to polyp can also lead to endometrial malignancy, logically resection of polyps could not prevent endometrial carcinoma.^[7]^ In our study, endometrial polyps consist of more than half (51.2%) pathological tissue, and endometrial lesions in TVS were only seen in 99 patients. The role of endometrial polyps’ resection for the prevention of malignancies deserves intensive prospective exploration.

Despite negative findings of the cut-off value of endometrial thickness in asymptomatic postmenopausal women, long-term follow-up and supervision are essential. In our median 45 months follow-up for patients with benign discoveries, cancer took place in only 1 of 31 cases (3.2%) of repeated thickened endometrium. As part results of the Prostate, Lung, Colorectal, and Ovarian Cancer Screening Trial, women with baseline endometrial thickness greater ≥5.0 mm in TVS were found to have an increased risk of endometrial (RR = 5.02, 95% CI = 0.96–26.36) carcinomas in models adjusted for menopausal hormone use and BMI.^[35]^ These discoveries guarantee the importance of long-term follow-up rather than invasive interventions.

There are several limitations to our study. First, its retrospective characteristic would give rise to recall bias and selection bias. The study lacked a complete review of medical records about metabolic syndrome, family history, and oral contraceptive use history, which all have a significant impact on the risk of endometrial cancer. Second, the standard of hysteroscopy may not be consistent during the 10 years, therefore resulting heterogeneity of pathological outcomes. Third, we lacked sufficient reports of complications with hysteroscopy, which need verification in prospective studies. Fourth, excluding patients with only D & C would miss considerable cases in a retrospective study, which is a potential source of bias.

In conclusion, among asymptomatic postmenopausal women with endometrial thickness ≥5 mm in TVS, risk of precancerous lesions or carcinoma was minimal. In such situation, detailed TVS could provide the most invaluable prediction for malignancies, although no definite cut-off value of xendometrial thickness existed to predict the nature of endometrial disease.

## Author contributions

**Contributors** LL conceived of the original idea for the study, interpreted results, carried out the statistical analysis, drafted the paper and is overall guarantor. ZL obtained ethical approval, contributed to the preparation of the data set, interpreted results and contributed to drafts of the paper. JL contributed to the study design, interpretation of results, and commented on drafts of the paper.

**Conceptualization:** Lei Li.

**Data curation:** Lei Li.

**Validation:** Lei Li.

**Writing – original draft:** Zhe Li.

**Writing – review and editing:** Lei Li.
